# The effect of nonanatomic positive medial cortical support on the reduction stability of unstable pertrochanteric proximal femur fractures: A cohort study

**DOI:** 10.1097/MD.0000000000042498

**Published:** 2025-05-16

**Authors:** Mohammad Shasho, Mohammad Al-Jawad, Muhammad Anas Kudsi, Hamza Khaiata, Ali Aswad, Yasser Iskandar

**Affiliations:** b University of Aleppo, Faculty of Medicine, Aleppo, Syria; a Al-Mowassat University Hospital, Damascus University, Damascus, Syria

**Keywords:** change criteria, cohort study, medial cortical support, pertrochanteric hip fractures

## Abstract

Older adults with osteoporosis often face challenges from pertrochanteric hip fractures, leading to suboptimal functional outcomes despite high union rates. For that reason, we conducted this study to evaluate the outcomes of these fractures, focusing on the impact of positive and negative medial cortical support on postoperative stability and recovery. Key factors influencing treatment success include bone quality, fracture configuration, and the effectiveness of fixation methods, with emerging criteria like the Chang reduction quality criteria offering improved assessment of stability. A retrospective analysis of 154 patients aged 60 and older with pertrochanteric fractures was conducted to evaluate the impact of fracture reduction quality on clinical outcomes. The study focused on pre-injury conditions, surgical techniques, postoperative recovery, and complications, highlighting the importance of effective management in this population. The study analyzed 154 patients with pertrochanteric fractures, categorizing them by medial cortex position, revealing significant differences in femoral neck–shaft angle loss and time to full weight-bearing among groups. Patients with positive medial cortical support experienced the least loss and achieved full weight-bearing faster than those with negative support. This study evaluates the clinical significance of the Chang reduction quality criteria in pertrochanteric fractures, highlighting its impact on neck–shaft angle changes and time to full weight-bearing among patients with varying medial cortical support. Results indicate that better reduction quality correlates with improved surgical outcomes and faster recovery, emphasizing the importance of achieving optimal fracture alignment. The Chang reduction quality criteria is a reliable and comprehensive tool for evaluating the quality of fracture reduction. Restoring normal hip biomechanics and allowing early mobilization are key to achieving a reduction with positive or neutral medial cortical support. This helps keep complications to a minimum and speeds up the patient’s recovery.

## 1. Introduction

In this study, we will use specific terms that are crucial for understanding the context of our research. Positive medial cortical support (PMCS) refers to the presence of supportive cortical bone on the inner side, which enhances fracture stability and reduces the risk of postoperative complications. Conversely, negative medial cortical support (NMCS) indicates a lack of adequate cortical support, increasing the likelihood of instability and adversely affecting treatment outcomes. These definitions are essential for comprehending the implications of our findings regarding hip fractures in older adults with osteoporosis.

Older adults with osteoporosis frequently experience pertrochanteric hip fractures^[1]^ which pose a significant challenge for orthopedic practitioners worldwide. Despite the high rates of fracture union, the functional outcomes tend to be unsatisfactory. A combination of factors, including medical comorbidities, patient adherence, fracture pattern, bone quality, and environmental factors, are believed to contribute to this poor result. Unfortunately, we cannot adequately address many of these factors at the time of the initial fracture presentation.^[2–4]^

In 1980, Kaufer identified 5 major factors that significantly impact the treatment outcomes of pertrochanteric hip fractures: the quality of the patient’s bone; the configuration and displacement of the fracture fragments are also important factors to consider; the choice of the fixation device; the accuracy of the fracture reduction; and the implant is precisely positioned within the femoral head.^[5]^

The commonly used fixation options for pretrochanteric fractures include both intramedullary and extramedullary devices. Intramedullary fixation, especially with proximal femoral nail antirotation and intramedullary nail with integrated compression and antirotation systems, is thought to be the best because it is more biomechanically stable. Regardless of the type of implant utilized, inadequate stability following fracture fixation may result in complications like limb shortening, ongoing hip pain, functional limitations, and the necessity for additional surgeries. Such negative consequences can considerably postpone a patient’s ability to mobilize and recover, highlighting the critical need for stable and reliable fixation in the treatment of these complex fractures.^[1,6,7]^

**Figure 1.**
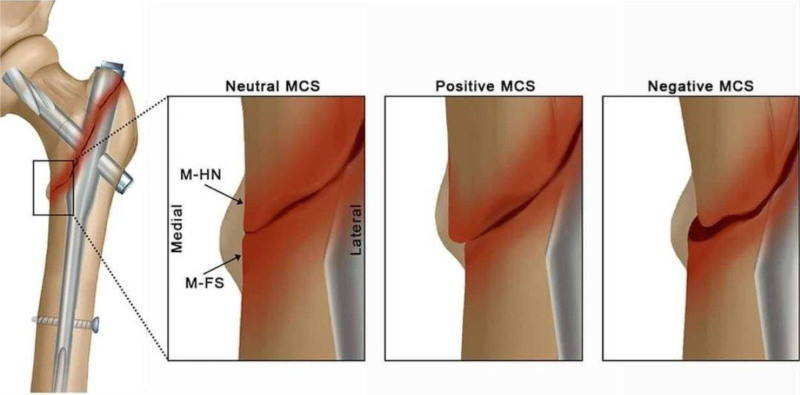
Explain the types of fractures and their mechanical classifications (neutral, positive, negative MCS).

Evaluating the quality of pretrochanteric fracture reductions is crucial in determining the stability and prognosis of these injuries. The most widely recognized criteria for this assessment are the Baumgaertner reduction quality criteria (BRQC), developed by Baumgaertner et al. Later, Chang et al proposed a new standard Table 1, the Chang reduction quality criteria (CRQC), which focuses on the presence or absence of PMCS and negative medial cortical support Figure 1. Studies have shown that the CRQC is more reliable than the BRQC in evaluating the stability of fracture fixation. This means that the CRQC might be a better and more complete way to judge the quality of pretrochanteric fracture reductions and guess how well treatment will likely work.^[1]^

**Table 1:** Chang criteria.

Item	Score
I. Alignment	
Anteroposterior view: normal or slight valgus neck–shaft angle	1*
Lateral view: <20° of angulation	1
II. Displacement†	
Anteroposterior view: neutral or positive medial cortical support	1
Lateral view: smooth anterior cortical contact	1
Reduction quality	
Excellent	4
Acceptable	2 or 3
Poor	0 or 1

MCS = medial cortical support.

*Slight valgus means a valgus of no more than 10°.^[4,14]^

†The displacement is less than half of the cortex thickness.

For that reason, we conducted this study to evaluate the outcomes of femoral neck fractures in older adults with osteoporosis, focusing on the impact of positive and negative medial cortical support on postoperative stability and recovery.

## 2. Patients and methods

### 2.1. Patient data collection

We performed a retrospective analysis on 154 consecutive patients, both men and women, who sustained pertrochanteric fractures between January 2022 and October 2022. All of these patients met the following criteria: The patients met the following criteria: (1) they were 60 years of age or older; (2) they were living at home prior to the injury; (3) they sustained hip fractures of non-pathological origin; (4) they were ambulatory without the use of assistive devices before the fracture occurrence; (5) they had no preexisting mental complications; and (6) they had specific fracture types classified as AO/OTA 31A2.2 and 31A2.3.

By choosing patients with these clear-cut traits, the study was able to focus on a homogeneous group of older adults who were functionally independent and living in the community before they sustained the pertrochanteric hip fractures. This cut down on potential confounding factors and made it possible to get a more accurate picture of how fracture reduction quality affects clinical outcomes.

It is important to note that patients with comorbidities, mental complications, and those who are not functionally independent typically require different rehabilitation protocols following surgery. Including these patients could introduce variability in outcomes, potentially skewing the results of our study. Therefore, we believe that excluding these groups allows for a more precise evaluation of the impact of fracture reduction quality on clinical outcomes.

We followed the patients for at least 1 year after the operation. We recorded information about the patient’s pre-injury condition, the specifics of the surgery and anesthesia, the postsurgical recovery, and the subsequent monitoring. Information gathered before injury encompassed the patient’s age, sex, and health status indicators such as hemoglobin (≥90 g/L) levels. Details of the surgical and anesthetic procedures included identifying the fracture type, measuring the duration and blood loss during surgery, recording any blood transfusions, and noting the length and kind of anesthesia administered. We tracked both medical and surgical complications postsurgery, including respiratory and urinary infections, confusion states, heart attacks, kidney failure, heart failure, strokes, blood clots in deep veins, peptic ulcers due to stress, and pressure sores. Postoperative management protocols included immediate X-ray imaging to establish a baseline assessment of the surgical site and implanted hardware. Follow-up imaging was performed at 2 weeks, 1.5 months, and 3 months to monitor healing and any changes in implant position or surrounding bone. This sequential imaging approach facilitated thorough evaluation and management of the patient’s postoperative recovery. We also assessed the patient’s ability to ambulate independently or with a single-point cane to determine full weight-bearing status.

### 2.2. Surgical technique and perioperative management

We treated hip fractures using the following steps: we laid the patients on their backs on a specialized table and administered either general or regional anesthesia. We used standard closed reduction techniques, such as spreading the legs apart, applying pulling force, and rotating inward, to align the fracture, and fluoroscopy confirmed the proper alignment. If closed methods failed to achieve alignment, we performed direct manipulation through the initial surgical cut.

We made an opening for the nail at the inner side of the greater trochanter’s tip and then reammed the upper portion of the bone marrow cavity. We chose the length and width of the short intramedullary nails based on the patient’s stature. The nail was used to disengage the head–neck fragment from the shaft, and pulling laterally on the nail jig helped to loosen the pieces, facilitating sagittal alignment with instruments like a bone hook or elongated tweezers.

For Gamma systems, we positioned a lag screw in the lower third of the femoral head in both the anterior–posterior view and the side view. We used a duple screw in fixed mode for distal locking.

After the surgery, we did not use any drains and administered blood transfusions if the hemoglobin levels dropped below 90 g/L. We administered cefuroxime for 2 days postsurgery as a preventive measure against infection, and used low-molecular-weight heparin to prevent blood clots. Notably, we avoided opioid pain relievers due to their potential to cause cognitive issues and breathing problems.

We have reported our work in accordance with the STROBE criteria.^[8]^

## 3. Data analysis

We conducted the data analysis using the statistical software package SPSS, version 27.0. We applied a post hoc test to compare the 3 groups based on the grade of medial cortical support.

We conducted nonparametric statistical tests after the Kolmogorov–Smirnov test revealed that the data did not follow a normal distribution, therefore, parametric statistical tests cannot be applied.

We performed a Kruskal–Wallis test to compare the means of the 3 groups (negative, neutral, and positive), revealing statistically significant differences between the group means. We then conducted a Mann–Whitney test to compare the means between each pair of groups. Statistical significance was defined as *P* < .05. There were no missing values in the data.

## 4. Results

The study included 154 patients, categorized based on their medial cortex position on X-rays. The majority (108 cases [70%]) had PMCS, followed by negative support (NMCS) at 32 cases (20.8%) and neutral support (NP) at 14 cases (9%).

We observed no significant statistical differences among the groups in terms of age, sex ratio, or the position of the lag screw or helical blade in the femoral head (TAD). Table 2 summarizes these details.

**Table 2:** The demographics and operative data.

	Positive medial cortical support	Neutral position	Negative medial cortical support
Cases	108 (70%)	14 (9%)	32 (20.8%)
Age	75.9 (62–95)	78.3 (70–92)	75.1 (62–95)
Male/female	47/61 (43.5%)	10/4 (71.4%)	10/22 (31.3%)
Fracture type (AO/OTA)			
31A2.2	36	4	12
31A2.3	72	10	20
TAD > 25 mm	6/108 (5.6%)	1/14 (7.1%)	2/32 (6.3%)
Quality 1*	81.666 ± 6.592	83.571 ± 8.187	82.0.31 ± 6.332

The table shows the distribution of the sample across the study groups. The table includes the percentage distribution of males and females, age, and fracture type in each group. The table also includes the quality 1 index. There was no statistically significant difference between the 3 study groups with regard to the quality 1 index (*P*-value = .600).

* Values are presented as mean ± standard deviation.

The initial postoperative measurement revealed an average neck–shaft angle of 134.5 ± 0.3°, which decreased to 130.7 ± 0.5° at the follow-up (after 3 months), indicating a varus change of 3.8 ± 0.35°. The mean loss of the femoral neck–shaft angle in the PMCS, NP, and NMCS groups was 1.4°, 4.3°, and 11.7°, respectively. The differences among these 3 groups were statistically significant. In other words, the PMCS group had the least statistical loss in neck–shaft angle, followed by the NP group, while the NMCS group experienced the greatest statistical loss.

In the whole group of patients, the mean time to achieve full weight-bearing was 5.46 ± 0.12 weeks. However, there was a significant difference in this time between the groups. NMCS patients took significantly longer compared to PMCS and NP patients. However, there was no significant difference between the NP and PMCS groups Table 3.

**Table 3:** Postoperative follow-up data.

	Positive medial cortical support	Neutral position	Negative medial cortical support
Postoperation neck–shaft angle	135.4 (130–141)	135 (130–138)	131.25 (124–135)
Angle after 3 mo follow-up	133.9 (128–140)	130.7 (127–136)	119.6 (115–123)
Loss of the angle (difference)*	1.4 ± 0.05	4.3 ± 0.66	11.7 ± 0.54
Timing of full weight-bearing (wk)*	4.8 ± 0.77	5.1 ± 0.22	7.7 ± 0.26
Quality 2	77.546 ± 6.502	70.357 ± 4.986	58.281 ± 7.026

The table shows the results of follow-up after surgical intervention. Statistical analysis showed a statistically significant difference when comparing the NMCS group with both the PMCS and neutral position groups in all variables included in the table (*P*-value < .001).

When comparing the PMCS and neutral position groups with each other, a statistically significant difference was found in angle after 3 months follow-up, loss of the angle and quality 2 index (*P*-value < .005), but there was no statistically significant difference for postoperation neck–shaft angle (*P*-value = .738) and timing of full weight-bearing (*P*-value = .316).

* Values are presented as mean ± standard deviation.

## 5. Discussion

The quality of fracture reduction is critical to achieving stable fixation. While many previous studies have included reduction quality as a confounding variable, this study focuses on the CRQC, which are a relatively new set of parameters for assessing the quality of reduction following fracture fixation. This study aimed to verify the clinical significance of the CRQC by comparing outcomes between patients with an excellent CRQC grade and those with lower CRQC grades. The CRQC is a complete evaluation tool that looks at different radiographic parameters to find out how well the fracture was reduced. By using the CRQC as the main outcome, this study shows how important it is to get a good reduction for the best surgical outcomes, instead of just looking at reduction quality as a secondary factor.

The surgical management of unstable pertrochanteric fractures prioritizes achieving anatomical reduction before determining the optimal placement of various recommended implants. Although aligning the posteromedial cortex is crucial for successful reduction, most current implants cannot secure the lesser trochanteric fragment. For these fractures, maintaining garden alignment and anteromedial contact between the femoral head–neck and shaft fragments is of utmost importance. However, a valgus position in fracture alignment does not equate to positive medial cortical support in fragment displacement.^[2]^

Recent studies have consistently demonstrated that the CRQC provide a more reliable assessment of fracture reduction quality compared to the traditional BRQC. The CRQC incorporates detailed parameters such as PMCS and NMCS, which enhance its predictive value for mechanical complications. In contrast, the BRQC tends to oversimplify the evaluation process, potentially overlooking critical aspects of fracture stability. These findings underscore the importance of adopting the CRQC in clinical practice to improve patient outcomes in trochanteric fractures.^[9]^

Discomfort and changes in biomechanics are key determinants influencing the quality of fracture reduction and subsequent mortality. The principal aim of surgery for pretrochanteric fractures is to facilitate early mobilization to minimize complications associated with prolonged bed rest and reduce mortality rates. Instability at the fracture site from not reducing it enough can also cause the limb to get shorter and biomechanical problems, especially a drop in the performance of the abductor muscles, which could lead to the same bad outcomes.^[10,11]^ Currently, the CRQC and the BRQ serve as the 2 main criteria for evaluating the quality of postoperative reduction in pretrochanteric fractures.^[9]^

The CRQC’s key advancement is the incorporation of PMCS and NMCS concepts. The CRQC stipulates that an acceptable reduction in anteroposterior (AP) views must fulfill 2 criteria: (1) a displacement less than the thickness of the bone cortex; and (2) neutral or positive medial cortical support. This definition of acceptable reduction excludes 2 scenarios: NMCS with displacement less than the fracture’s cortical thickness or PMCS with displacement exceeding the fracture’s cortical thickness. For side views, the CRQC needs a smooth anterior cortex, with movement being less than half of the thickness of the bone cortex. This shows how important strong anterior cortical support is. Presently, most internal fixation devices do not stabilize small trochanter fragments; hence, the CRQC does not mandate posterior cortical alignment explicitly. For several reasons, we deem the CRQC more reliable than the Baumgaertner reduction quality (BRQ). Primarily, the BRQ may overlook certain details; for instance, it groups nonalignment into 3 potential scenarios—poor alignment solely on AP views, solely on lateral views, or on both—and fails to differentiate among them. Conversely, the CRQC employs a more comprehensive 4-point scoring system that preserves greater detail.^[2,9,12,13]^

Secondly, the CRQC judiciously employs PMCS and NMCS concepts. PMCS provides cortical support between the primary fracture fragments, preventing the femoral head and neck fragments from moving further laterally. On the other hand, NMCS is marked by the interaction between the proximal bone cortex and the distal spongy bone. The proximal bone mass embeds into the low-density central region, which allows the femoral head and neck fragments to move laterally even more, which could cause the internal fixation to fail. BRQ, on the other hand, says that an adequate AP view reduction is a displacement of <4 mm. However, both PMCS and NMCS can happen with a displacement of <4 mm, and each can have different clinical outcomes.^[9]^

Nevertheless, the CRQC offers a viable resolution to this issue. Third, using 1 or half cortical thickness as a measure of displacement is more useful than giving a precise distance of 4 mm because it allows for direct visual assessment using C-arm fluoroscopy during surgery without the need for special tools.

Previous studies have consistently demonstrated that increased age and the presence of multiple comorbidities are independent risk factors that negatively impact older adults’ survival following hip fractures. Cui et al discovered a positive correlation between mortality rates and advancing age in this patient population.^[14]^ Lei et al also looked back at data from 1057 hip fracture patients aged 60 and up and found that having other health problems was linked to a higher 5-year death rate after surgery.^[15]^ Our study found that mortality increased with age and the number of comorbidities during the total follow-up.

The recommendations for achieving a satisfactory quality of fracture reduction for pretrochanteric hip fractures include the following key elements: position the reduced fracture in a slight valgus alignment with positive medial cortical support in the AP view, ensuring continuity and contact between the medial cortices of the proximal and distal fragments. The sagittal (lateral) view should optimize the central axial alignment of the reduced fracture, positioning the nail or implant centrally within the femoral head and neck, and ensuring smooth contact between the anterior cortices of the proximal and distal fragments, without any step-offs or displacement. These specific radiographic parameters are very important for making sure a high-quality fracture reduction, which is needed to restore the normal biomechanics of the hip joint and make early mobilization and rehabilitation easier. This will ultimately improve surgical outcomes and lower the risk of complications related to poor reduction quality.

Our study examined changes in the neck–shaft angle and time to full weight-bearing among patients treated with different surgical techniques for hip fractures. At the 3-month follow-up, the initial neck–shaft angle of 134.5° decreased to 130.7°, representing a 3.8° varus change. Comparing surgical groups, the PMCS group had the least statistical loss (1.4°), followed by NP (4.3°) and NMCS (11.7°), with significant differences between the groups.

The mean time to full weight-bearing was 5.46 weeks, but this differed significantly between groups. NMCS patients took longer compared to PMCS and NP, with no difference between NP and PMCS.

One significant limitation of our study is the involvement of multiple surgeons in the procedures for the patients included in the analysis. This variability in surgical experience makes it challenging to control for the impact of individual surgeon expertise on the surgical outcomes. As a result, the influence of surgeon experience on recovery time and weight-bearing capacity may not be adequately addressed.

Additionally, the follow-up period of 1 year is relatively short and may not be sufficient to capture long-term complications, reoperations, or significant differences in mortality rates. We acknowledge that future studies should consider implementing a longer follow-up period to provide a more comprehensive assessment of recovery and postoperative outcomes.

## 6. Conclusion

Our study found that patients with NMCS had a greater decrease in neck–shaft angle and took longer to achieve full weight-bearing compared to patients with positive or neutral support. This suggests that the NMCS patient group has the worst postoperative outcomes. Therefore, avoiding reductions with negative cortical support is crucial.

## Acknowledgments

I would like to express my heartfelt gratitude to Mohamad Ali Keblawi from Qumrah Research Lab, for his tremendous support and guidance. His assistance in direction and review was crucial in helping me complete this work in the best possible way.

## Author contributions

**Data curation:** Ali Aswad.

**Methodology:** Ali Aswad, Mohammad Al-Jawad

**Supervision:** Mohammad Shasho, Mohammad Al-Jawad, Yasser Iskandar.

**Writing – original draft:** Mohammad Al-Jawad, Muhammad Anas Kudsi, Hamza Khaiata.

**Writing – review & editing:** Mohammad Al-Jawad.

## References

[1] He M Liu J Deng X Zhang X. The postoperative prognosis of older intertrochanteric fracture patients as evaluated by the Chang reduction quality criteria. BMC Geriatr. 2022;22:2. 36457103 10.1186/s12877-022-03641-z PMC9717473

[2] Chang SM Zhang YQ Ma Z Li Q Dargel J Eysel P. Fracture reduction with positive medial cortical support: a key element in stability reconstruction for the unstable pertrochanteric hip fractures. Arch Orthop Trauma Surg. 2015;135:811–8. 25840887 10.1007/s00402-015-2206-x PMC4436685

[3] Kokoroghiannis C Aktselis I Deligeorgis A Fragkomichalos E Papadimas D Pappadas I. Evolving concepts of stability and intramedullary fixation of intertrochanteric fractures—a review. Injury. 2012;43:686–93. 21752370 10.1016/j.injury.2011.05.031

[4] Tsang STJ Aitken SA Golay SK Silverwood RK Biant LC. When does hip fracture surgery fail? Injury. 2014;45:1059–65. 24794618 10.1016/j.injury.2014.03.019

[5] Mechanics of the treatment of hip injuries – PubMed. [cited June 9, 2024]. https://pubmed.ncbi.nlm.nih.gov/7371269/.

[6] Shen L Zhang Y Shen Y Cui Z. Antirotation proximal femoral nail versus dynamic hip screw for intertrochanteric fractures: a meta-analysis of randomized controlled studies. Orthop Traumatol Surg Res. 2013;99:377–83. 23707739 10.1016/j.otsr.2012.12.019

[7] Evaniew N Bhandari M. Cochrane in CORR®: intramedullary nails for extracapsular hip fractures in adults (review). Clin Orthop Relat Res. 2015;473:767–74. 25560962 10.1007/s11999-014-4123-7 PMC4317456

[8] STROBE – Strengthening the reporting of observational studies in epidemiology. [cited July 9, 2024]. https://www.strobe-statement.org/.

[9] Mao W Ni H Li L. Comparison of Baumgaertner and Chang reduction quality criteria for the assessment of trochanteric fractures. Bone Joint Res. 2019;8:502–8. 31728190 10.1302/2046-3758.810.BJR-2019-0032.R1 PMC6825041

[10] Gilat R Lubovsky O Atoun E Debi R Cohen O Weil YA. Proximal femoral shortening after cephalomedullary nail insertion for intertrochanteric fractures. J Orthop Trauma. 2017;31:311–5. 28538452 10.1097/BOT.0000000000000835

[11] Macdonald H Brown R Gronager M Close J Fleming T Whitehouse M. Quality of fracture reduction is associated with patient survival at one year, but not 30 days, following trochanteric hip fracture fixation. A retrospective cohort study. Injury. 2022;53:1160–3. 35058064 10.1016/j.injury.2021.12.048

[12] Kozono N Ikemura S Yamashita A Harada T Watanabe T Shirasawa K. Direct reduction may need to be considered to avoid postoperative subtype P in patients with an unstable trochanteric fracture: a retrospective study using a multivariate analysis. Arch Orthop Trauma Surg. 2014;134:1649–54. 25260901 10.1007/s00402-014-2089-2

[13] Jiamton C Boernert K Babst R Beeres FJP Link BC. The nail–shaft-axis of the proximal femoral nail antirotation (PFNA) is an important prognostic factor in the operative treatment of intertrochanteric fractures. Arch Orthop Trauma Surg. 2018;138:339–49. 29256184 10.1007/s00402-017-2857-x

[14] Cui Z Feng H Meng X. Age-specific 1-year mortality rates after hip fracture based on the populations in mainland China between the years 2000 and 2018: a systematic analysis. Arch Osteoporos. 2019;14. 10.1007/s11657-019-0604-3 PMC6535151 31129721

[15] Jiang L Chou ACC Nadkarni N. Charlson comorbidity index predicts 5-year survivorship of surgically treated hip fracture patients. Geriatr Orthop Surg Rehabil. 2018;9. 10.1177/2151459318806442 PMC6249653 30479849

